# Exploring Factors Related to Social Isolation Among Older Adults in the Predementia Stage Using Ecological Momentary Assessments and Actigraphy: Machine Learning Approach

**DOI:** 10.2196/69379

**Published:** 2025-06-23

**Authors:** Bada Kang, Min Kyung Park, Jennifer Ivy Kim, Seolah Yoon, Seok-Jae Heo, Chaeeun Kang, SungHee Lee, Yeonkyu Choi, Dahye Hong

**Affiliations:** 1 Mo-Im Kim Nursing Research Institute Yonsei University College of Nursing Seoul Republic of Korea; 2 Institute for Innovation in Digital Healthcare Yonsei University Seoul Republic of Korea; 3 Department of Nursing Graduate School of Yonsei University Seoul Republic of Korea; 4 Biostatistics Collaboration Unit Department of Biomedical Systems Informatics Yonsei University College of Medicine Seoul Republic of Korea; 5 Department of Nursing Yonsei University College of Nursing Seoul Republic of Korea; 6 BRFrame Inc Seoul Republic of Korea; 7 College of Nursing and Brain Korea 21 FOUR Project Yonsei University Seoul Republic of Korea

**Keywords:** social isolation, social interaction, loneliness, aged, machine learning, ecological momentary assessment, actigraphy, predementia

## Abstract

**Background:**

As the global population ages, the economic burden of dementia continues to rise. Social isolation—which includes limited social interaction and feelings of loneliness—negatively affects cognitive function and is a significant risk factor for dementia. Individuals with subjective cognitive decline and mild cognitive impairment represent predementia stages in which functional decline may still be reversible. Therefore, identifying factors related to social isolation in these at-risk groups is crucial, as early detection and intervention can help mitigate the risk of further cognitive decline.

**Objective:**

This study aims to develop and validate machine learning models to identify and explore factors related to social interaction frequency and loneliness levels among older adults in the predementia stage.

**Methods:**

The study included 99 community-dwelling older adults aged 65 years and above in the predementia stage. Social interaction frequency and loneliness levels were assessed 4 times daily using mobile ecological momentary assessment over a 2-week period. Actigraphy data were categorized into 4 domains: sleep quantity, sleep quality, physical movement, and sedentary behavior. Demographic and health-related survey data collected at baseline were also included in the analysis. Machine learning models, including logistic regression, random forest, Gradient Boosting Machine, and Extreme Gradient Boosting, were used to explore factors associated with low social interaction frequency and high levels of loneliness.

**Results:**

Of the 99 participants, 43 were classified into the low social interaction frequency group, and 37 were classified into the high loneliness level group. The random forest model was the most suitable for exploring factors associated with low social interaction frequency (accuracy 0.849; precision 0.837; specificity 0.857; and area under the receiver operating characteristic curve 0.935). The Gradient Boosting Machine model performed best for identifying factors related to high loneliness levels (accuracy 0.838; precision 0.871; specificity 0.784; and area under the receiver operating characteristic curve 0.887).

**Conclusions:**

This study demonstrated the potential of machine learning–based exploratory models, using data collected from mobile ecological momentary assessment and wearable actigraphy, to detect vulnerable groups in terms of social interaction frequency and loneliness levels among older adults with subjective cognitive decline and mild cognitive impairment. Our findings highlight physical movement as a key factor associated with low social interaction frequency, and sleep quality as a key factor related to loneliness. These results suggest that social interaction frequency and loneliness may operate through distinct mechanisms. Ultimately, this approach may contribute to preventing cognitive and physical decline in older adults at high risk of dementia.

**International Registered Report Identifier (IRRID):**

RR2-10.1177/20552076241269555

## Introduction

The annual global societal cost of dementia was US $1313.4 billion in 2019, representing an enormous economic burden [1]. With the global dementia population projected to increase from 57.4 million in 2019 to 152.8 million by 2050 [2], this burden is expected to rise substantially [3]. To alleviate it, it is crucial to identify risk factors and implement preventive interventions at the most effective times across the dementia spectrum [4].

Social isolation is one of the risk factors for dementia among older adults [5-7]. It is a complex concept that primarily refers to the objective aspect of low frequency of social interactions but is also closely related to the subjective experience of loneliness [8]. While social interaction refers to the frequency of contact with others [9], loneliness is a subjective and negative feeling caused by a lack of social networks [10]. Experiencing social isolation has been associated with reduced gray matter volume in the memory-related hippocampus [7,11], potentially increasing the risk of developing dementia. As a modifiable risk factor for dementia [12-14], it is essential to identify and address aspects of social isolation among at-risk individuals through early detection.

Early detection of social isolation in at-risk groups for dementia, such as individuals with subjective cognitive decline (SCD) and mild cognitive impairment (MCI), may enhance the effectiveness of dementia prevention efforts. SCD and MCI represent the preclinical and prodromal stages of dementia, respectively [15]. SCD refers to a sustained, self-perceived decline in cognitive abilities compared with a previous level, without objective cognitive impairment [16]. By contrast, MCI differs from SCD in that it involves objective cognitive impairment [16]. Compared with individuals with normal cognition, the risk of dementia is more than twice as high for those with SCD [17], and over 23 times higher for those with MCI, indicating that SCD and MCI are vulnerable groups in the progression to dementia [18]. However, both SCD and MCI represent stages where recovery of cognitive function and delayed progression are possible through prompt identification of high-risk individuals and reduction of modifiable risk factors [19,20]. Thus, the SCD and MCI stages may represent an optimal window to implement interventions aimed at improving social interaction and preventing loneliness—both modifiable risk factors for dementia [21]. Additionally, with an estimated 315 million people globally affected by SCD and 69 million by MCI in 2020 [22], addressing social isolation at these stages through early detection could have significant public health implications for dementia prevention.

Ecological momentary assessment (EMA) enables real-time self-reported data collection in everyday environments, reducing recall bias and allowing for more accurate measurement [23]. Previous studies have primarily relied on retrospective methods to assess social interaction and loneliness [24,25]. However, these methods are subject to recall bias and typically involve single-time assessments, which may not adequately capture actual experiences. This limitation is especially pronounced in populations with memory impairments, such as individuals with SCD and MCI [26]. EMA offers several advantages over traditional measurement methods. Recent studies have evaluated EMA as a more time-sensitive tool for capturing the characteristics of social isolation [26], highlighting its potential to provide a more nuanced understanding of this phenomenon [27]. These strengths underscore EMA’s suitability as a reliable method for assessing social interaction and loneliness, particularly in cognitively vulnerable populations.

Additionally, actigraphy continuously and noninvasively records data on activity and sleep in real time during everyday activities, minimizing recall bias and enabling objective measurement [28-30]. Given these advantages, actigraphy has proven useful in predicting social isolation [31]. However, a retrospective study that used regression models to explore the relationship between social interaction, loneliness, and sleep among older adults in the United States [31] had limitations in capturing sleep characteristics. This was due to the use of only 3 sleep variables—total sleep time (TST), sleep efficiency, and wake after sleep onset (WASO)—measured via actigraphy over a relatively short period (3 days). The previous study has limited applicability to older adults with cognitive impairment, as this population was not included. In addition, although increased physical activity has the potential to promote contact with others—thereby enhancing social interactions and reducing loneliness—prior research has primarily focused on sleep-related variables, failing to fully consider other factors associated with social isolation [32,33]. Therefore, applying objectively assessed sleep and physical activity data collected through real-time assessment methods would not only enhance data reliability but also provide a more comprehensive understanding of social isolation.

Machine learning (ML) is a powerful tool for identifying patterns and predicting outcomes in large data sets, including those collected over extended periods [34]. ML is particularly valuable for processing actigraphy data, which includes metrics such as physical activity and sleep, and shows significant promise for predictive purposes [31,35,36]. Given its capacity to handle large data sets, applying ML to mobile EMA data, actigraphy-based sleep and physical activity data, and survey data represents a novel approach to accurately predict social isolation among at-risk groups. Therefore, the objective of our study was to develop and validate models to explore factors related to 2 aspects of social isolation—social interaction frequency and levels of loneliness—among older adults in the predementia stage.

## Methods

### Design

This study used a prospective observational design to build models exploring factors associated with social interaction frequency and levels of loneliness among older adults with SCD or MCI.

### Ethics Considerations

Ethical approval for this study was obtained from the Yonsei University Health System Severance Hospital Institutional Review Board (approval number 4-2022-0637). All participants voluntarily provided written informed consent before enrollment and were free to withdraw at any time without penalty. Anonymized participant data were collected and stored on an encrypted server. This manuscript uses data from the first wave of a larger 3-wave study, for which the study protocol has been previously published [37].

### Participants and Settings

#### Inclusion and Exclusion Criteria

Older adults with SCD or MCI were recruited from the Dementia Relief Center and a community service center in Seoul, Korea. The Dementia Relief Center houses an outpatient clinic specializing in the early diagnosis and care management of dementia, whereas the community service center serves as a space for older adults to engage in recreational and wellness programs, regardless of dementia diagnosis. The common inclusion criteria for study participants were (1) age over 65 years, (2) ability to use a smartphone, and (3) ability to respond to momentary questionnaires via a mobile app. The recruitment criteria were specifically tailored to the characteristics of SCD and MCI. The inclusion criteria for each group are listed in the following sections.

#### SCD Group

All participants with SCD answered “yes” to the question, “Do you think your memory has worsened compared to before?” Considering the different characteristics of the Dementia Relief Center and the community service center, tailored recruitment criteria were applied for each setting. Participants recruited from the Dementia Relief Center had no history of MCI or dementia, as reported by a nurse. Those recruited from the community service center self-reported no history of MCI or dementia and had a score of 24 or higher on the Korean Mini-Mental State Examination, second edition (K-MMSE-2) [38-40].

#### MCI Group

The MCI group consisted of participants who had been clinically diagnosed with MCI by a physician at the Dementia Care Center. To ensure their cognitive function was not consistent with severe cognitive impairment, individuals with MCI were additionally screened using the K-MMSE-2 and were required to score 18 or higher.

Participants were excluded if they met any of the following criteria: (1) illiteracy; (2) diagnosis of neurological diseases such as epilepsy, stroke, Parkinson disease, or other forms of brain damage; (3) diagnosis of psychiatric disorders such as schizophrenia or bipolar disorder; or (4) undergoing treatment for critical illnesses such as chemotherapy, having severe cardiovascular disease, or a history of substance abuse (including narcotics or alcohol) within the past 3 years.

### Procedure

Participant recruitment and data collection were conducted through in-person visits from October 2022 to November 2023. These activities were carried out by a registered nurse enrolled in a master’s program (SY) and undergraduate nursing students, including CK. A master’s-level RN (DH), serving as the research coordinator, trained the research team in recruitment and data collection procedures according to the study protocol. Recruitment was primarily conducted by RNs, who verified participants’ eligibility, explained the purpose of the study, and obtained informed consent. Undergraduate nursing students, serving as research assistants, supported the process by installing the mobile EMA app on participants’ phones and registering their information in the actigraphy program. After the 2-week study period, the research team met with participants in person to collect actigraphy and survey data and to remove the EMA application from their smartphones.

### EMA Features

Participants recorded their social interaction frequency and levels of loneliness in real time using a mobile EMA app on their smartphones. These variables were assessed 4 times daily, based on the Time-Use Survey from Statistics Korea [41]: night (9 PM-9 AM), morning (9 AM-2 PM), afternoon (2 PM-6 PM), and evening (6 PM-9 PM).

Social interaction frequency was measured using a 5-point Likert scale (0=no contact to 4=four or more times), based on the frequency of interpersonal contact [42], including in-person meetings, phone calls, and video calls. Although validation studies of single-item EMA measures of social interaction frequency in older adults are limited, we assessed the reliability of this item using the intraclass correlation coefficient (ICC), which was 0.33, indicating fair agreement [43].

Levels of loneliness, as a subjective feeling, were assessed using a 5-point Likert scale ranging from “not lonely at all” to “extremely lonely.” A previous study using a similar single-item EMA approach to assess momentary loneliness among older adults reported an ICC of 0.75 [44]. In our study, the ICC for the loneliness item was 0.56, indicating moderate agreement [43] and supporting the reliability of this measure for repeated assessments.

### Survey Data

We utilized a self-reported survey to collect demographic and health-related data. Demographic variables included sex, age, educational level, marital status, household type, subjective economic status, previous employment status, and duration of previous employment. Health-related variables included medical history, vision impairment, hearing impairment, subjective health status, number of medications, cognitive status, functional status, and psychological status.

### Cognitive Status and Functional Status

In this study, the K-MMSE-2 [39,40] was used to assess cognitive functioning. Scores range from 0 to 30, with lower scores indicating poorer cognitive function. We also used the Korean version of the Subjective Cognitive Decline Questionnaire (SCD-Q) to measure self-perceived cognitive decline [45]. Scores range from 0 to 24, with higher scores indicating a greater subjective perception of cognitive decline [46].

We utilized the Korean Instrumental Activities of Daily Living (K-IADL) to assess functional status. Among the 10 items in the K-IADL, 3 items are scored on a scale of 1-3 points, while the remaining items are scored on a scale of 1-4 points. Higher scores indicate a greater level of dependency [47]. Participants were categorized into 2 groups: those reporting dependency on 1 or more items were classified as dependent, while those independent on all items were classified as independent [48]. We also used the Frailty Phenotype Questionnaire to assess frailty among community-dwelling older adults. Scores range from 0 to 5, with 0 indicating robust status, 1-2 indicating prefrailty, and 3-5 indicating frailty [49].

### Psychological Status

We used the Korean version of the Short Form Geriatric Depression Scale (SGDS-K) to measure depression [50]. The SGDS-K ranges from 0 to 15, with higher scores indicating greater depressive symptoms. Additionally, we used the Korean version of the Geriatric Anxiety Inventory (K-GAI) [51] to assess anxiety levels. The K-GAI ranges from 0 to 20, with higher scores indicating higher levels of anxiety. We also utilized the Korean version of the Mild Behavioral Impairment Checklist (MBI-C) [52] to assess symptoms of mild behavioral impairment (MBI). MBI is a concept encompassing changes in motivation, affect, impulse control, social appropriateness, and perception or thought content [53], and is associated with the transition from a nondementia state to dementia [54]. The Korean version of the MBI-C comprises domains corresponding to the 5 core components of MBI. The presence of MBI was determined by any score greater than 0, assessed from both global and domain-specific perspectives. Additionally, MBI severity was evaluated using summed scores, which were calculated separately as global total scores and domain-specific total scores.

### Actigraphy

Participants wore a wrist-worn Actiwatch (Actiwatch Spectrum PRO; Philips Respironics) continuously for 2 weeks (24 hours a day). They were instructed to wear the device throughout the study period, except during unavoidable situations such as showering or when experiencing discomfort. The Actiwatch is a reliable and effective device for measuring sleep and physical activity [55] and has been widely used in studies involving older adults [36,56]. We used the Actiwatch Spectrum PRO, which is equipped with an accelerometer that samples data 32 times per second. It was configured with a 30-second epoch length and a threshold value of 40 to collect sleep and physical activity data. Raw data were exported using Actiware software (version 6.1.2.1; Philips Respironics). The Actiware algorithm is commonly used for actigraphy data extraction and is well-regarded for its performance [57].

If activity counts were below the threshold value of 40, they were categorized as sleep, while counts exceeding 40 were classified as wake [30]. Additionally, immobile and mobile states were defined by Actiware based on the level of activity generated during each 30-second epoch. Activity was classified as mobile if the activity count was 2 or more within the epoch, and immobile if the activity count was less than 2 [29].

### Sleeping Features

We generated 5 sleep features: TST (ie, the sum of the lengths of sleep bouts), number of sleep bouts (ie, the count of continuous sleep bouts), WASO (ie, the count of awakenings during sleep bouts, where sleep/wake is scored as 1), sleep efficiency (ie, TST divided by the sum of TST and WASO, multiplied by 100), and fragmentation index (ie, the sum of percent mobile and percent 1-minute immobile bouts, divided by the number of immobile bouts for the given interval). Among these 5 features, TST was classified as an indicator of sleep quantity, whereas the number of sleep bouts, WASO, sleep efficiency, and fragmentation index were classified as sleep quality indicators.

### Physical Activity Features

We derived 3 physical activity features: total activity counts (the sum of all activity counts), average mobile bouts (mobile time divided by the number of mobile bouts), and immobile time (the sum of intervals with activity counts of 2 or fewer). Total activity counts and average mobile bouts were classified as indicators of physical movement, whereas immobile time (minutes) was categorized as an indicator of sedentary behavior.

### Data Processing

For survey data preprocessing, we standardized continuous variables using a Min-Max scaler and applied one-hot encoding to categorical variables. For actigraphy data, temporal pattern characteristics were extracted using an autoencoder. An autoencoder is a neural network composed of an encoder and a decoder, which enables automatic feature learning from unlabeled data [58]. Eight actigraphy features were extracted across the 4 designated periods.

Because of challenges in completing all 4 mobile EMA assessments on the first and last days of the study—resulting from variations in participants’ start and end times—we used data collected from the second to the thirteenth day. This approach ensured consistency and reliability in the analysis and enabled precise time-based analysis and pattern identification. Participants were allowed to correct their responses if they initially submitted incorrect entries via the mobile EMA. In cases of duplicate responses at the same time point, the most recent entry was considered the corrected value and used for analysis. For missing responses, values within a 1-hour window were used to fill in the gaps.

**Figure 1:**
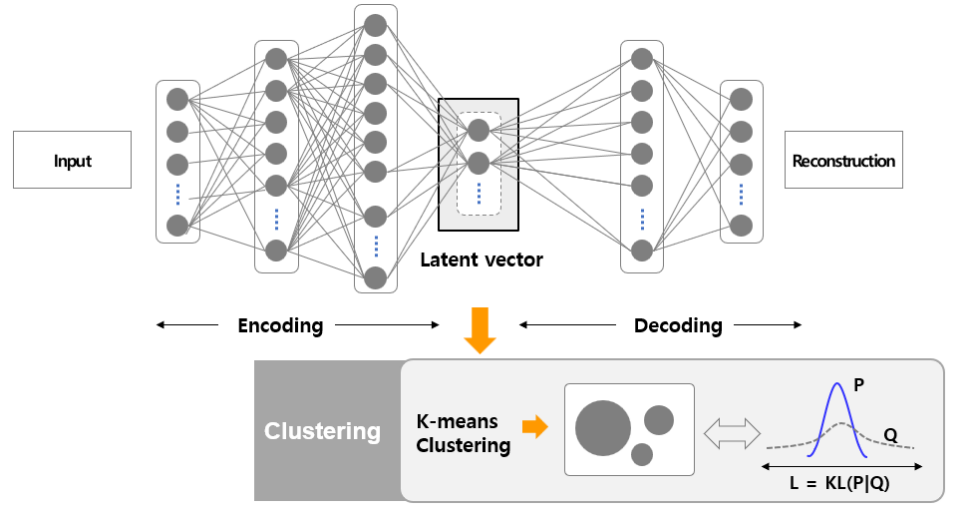
Deep embedding used for clustering data into 3 clusters.

The mobile EMA scores for social interaction frequency and loneliness levels are inherently subjective, with individual differences in interpreting neutral points. These variations made it challenging to determine whether a participant exhibited low social interaction frequency or high levels of loneliness based solely on changes in EMA scores over the study period. To address this issue, we applied Deep Embedding Clustering (DEC) [59], a deep learning–based unsupervised learning method, to group participants into a small number of clusters using dissimilarity-based analysis of their EMA responses (Figure 1). A detailed description of the DEC model architecture, hyperparameter settings, and training procedure is provided in Multimedia Appendix 1. DEC processes 48 EMA responses each for social interaction frequency and loneliness, embedding them into vectors via an autoencoder. These compressed vectors were then used for clustering.

The optimal number of clusters was determined by testing cluster counts ranging from 2 to 10, using 2 methods: (1) identifying the point where the silhouette coefficient is maximized [60], and (2) applying the elbow method, which involves selecting the point where the within-cluster sum of squares sharply declines [61]. Priority was given to the silhouette coefficient, while the elbow method was used as a supplementary approach to confirm the selection. Based on the results presented in Multimedia Appendix 2, 3 clusters were identified as optimal for social interaction levels, as this was the point where the silhouette coefficient reached its maximum. For the level of loneliness, although the silhouette coefficient was maximized at 2 clusters, the difference between 2 and 3 clusters was minimal. Moreover, 3 clusters corresponded to the point where the within-cluster sum of squares showed a sharp decline. Based on this, 3 clusters were selected as the optimal number. Consequently, we determined that 3 clusters were optimal for both social interaction frequency and loneliness levels.

**Figure 2:**
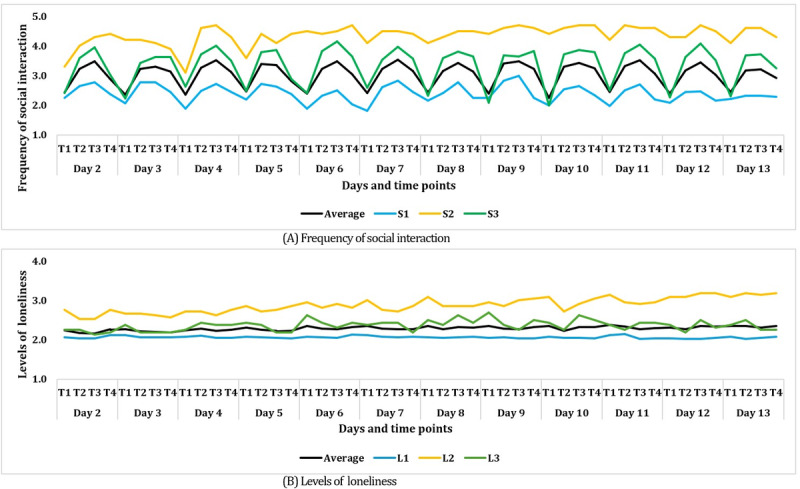
The classification of social interaction frequency and levels of loneliness of the 3 clusters. (A) Average graph of mobile EMA responses for the social interaction frequency of the 3 clusters. (B) Average graph of mobile EMA responses for the loneliness levels of 3 clusters. EMA: ecological momentary assessment.

The derived clusters were labeled S1, S2, and S3 for social interaction frequency, and L1, L2, and L3 for loneliness. To explore factors associated with low social interaction frequency and high levels of loneliness—the primary objectives of this study—these clusters were reclassified into binary groups. For this reclassification, we analyzed the mean EMA scores across the 4 daily time points during the study period (Figure 2) and examined the dispersion of participants’ embedding vectors generated by the Autoencoder (Multimedia Appendix 3). Based on these evaluations, we redefined the 3 clusters into 2 binary groups to classify participants as having either low or high social interaction frequency and loneliness.

### Exploratory Modeling

We used leave-some-out cross-validation to construct the training and validation data sets. The complete data set of 99 samples was divided into 10 folds using stratified sampling, separately for low social interaction frequency and high levels of loneliness. Each fold was used once for validation, and the performance metrics from all 10 iterations were aggregated for a comprehensive evaluation. Subsequently, we applied supervised learning models, including logistic regression, random forest (RF), Gradient Boosting Machine (GBM), and Extreme Gradient Boosting (XGBoost), to identify factors associated with social interaction frequency and loneliness.

**Figure 3:**
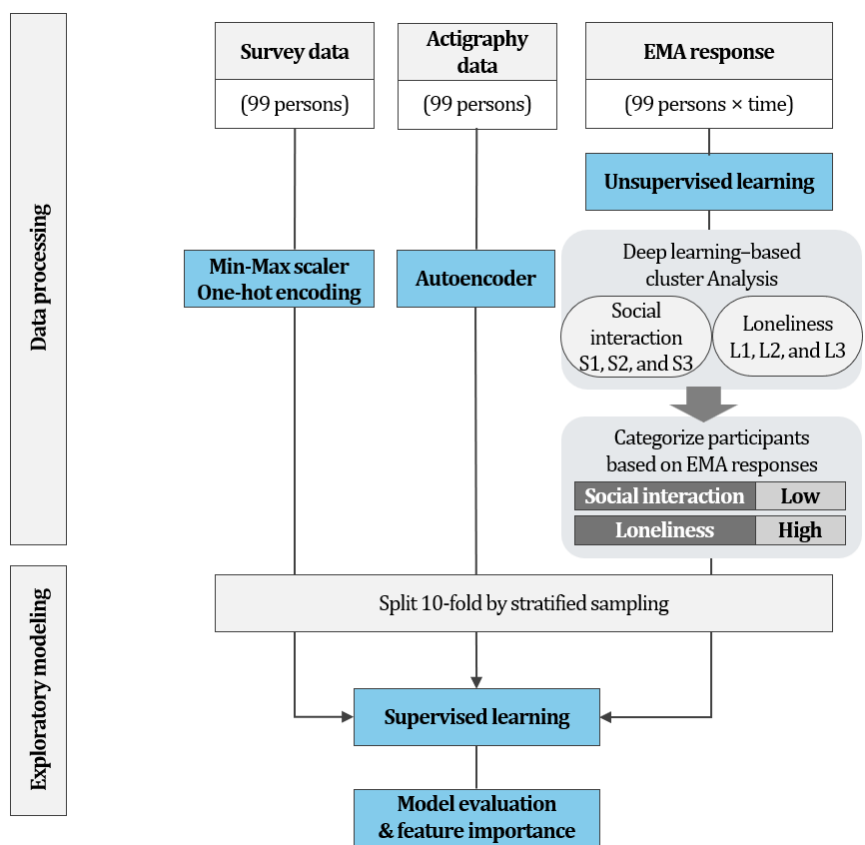
The sequential steps involved data processing and exploratory modeling. EMA: Ecological Momentary Assessment.

The relative importance of the variables associated with the models was assessed using the Permutation Feature Importance algorithm [62] (Figure 3).

### Statistical Analysis

We conducted descriptive analyses to examine the general characteristics of the study population. Continuous variables are reported as means with SDs, and categorical variables are presented as frequencies and percentages. To assess differences in general characteristics between the 2 groups for social interaction frequency and level of loneliness, chi-square tests and independent 2-tailed *t* tests were performed. The ML models were evaluated using the area under the receiver operating characteristic curve (AUC), accuracy, precision, specificity, and *F*_1_-score. Descriptive analyses were conducted using SAS version 9.4 (SAS Institute Inc), and a 2-sided *P* value of <.05 was considered statistically significant. ML analyses were conducted using Python version 3.9 (Python Software Foundation).

## Results

### Overview

A total of 145 participants were initially recruited for this study. Of these, 18 participants withdrew consent, and 1 participant was excluded due to device loss. Additionally, 27 individuals were excluded for having mobile EMA records covering fewer than 13 days, in order to ensure sufficient data for the 12-day analysis period and maintain data completeness. As a result, the final analysis included 99 participants—67 with SCD and 32 with MCI.

### Social Interaction and Loneliness Group Categorization

Based on the results of the DEC, social interaction frequency was categorized into 2 groups: the low group, which had below-average social interaction frequency (<average; S1), and the high group, which had above-average frequency (>average; S2 and S3). Similarly, levels of loneliness were divided into 2 groups: the low group, with below-average loneliness (<average; L1), and the high group, with above-average loneliness (≥average; L2 and L3; Figure 2).

As shown in Multimedia Appendix 3, visual inspection confirmed that the 2 groups were appropriately classified. For social interaction frequency, 43 participants were classified into the low group (S1), while 10 and 46 participants were classified into the high group (S2 and S3, respectively). For loneliness, 62 participants were classified into the low group (L1), while 21 and 16 participants were classified into the high group (L2 and L3, respectively).

### General Characteristics of Participants

Of the 99 participants, 62 (63%) were female. The mean age of all participants was 76.8 (SD 6.0) years. The mean age was 78.4 (SD 5.7) years in the low social interaction frequency group and 75.6 (SD 5.9) years in the high social interaction frequency group. Table 1 presents the baseline characteristics according to levels of social interaction. The low social interaction frequency group included 43 (43%) individuals. Compared with the high social interaction group, this group had significantly higher scores on the Korean version of the MBI-C (mean 9.9, SD 10.1 vs mean 5.1, SD 6.2; *P*=.01).

**Table 1:** Baseline characteristics of participants by social interaction frequency (N=99).

Variables		Low (n=43)	High (n=56)	*P* value
**Predementia stage, n (%)**				.63
	SCD^a^	28 (65)	39 (70)	
	MCI^b^	15 (35)	17 (30)	
**Sex, n (%)**				.004
	Men	23 (53)	14 (25)	
	Women	20 (47)	42 (75)	
**Age (years), n (%)**				.13
	<70	3 (7)	12 (21)	
	70-74	6 (14)	11 (20)	
	75-79	17 (40)	14 (25)	
	≥80	17 (40)	19 (34)	
**Educational level, n (%)**				.66
	≤Elementary school	15 (35)	14 (25)	
	Middle school	8 (19)	9 (16)	
	High school	9 (21)	15 (27)	
	≥College	11 (26)	18 (32)	
**Marital status, n (%)**				.45
	Married	30 (70)	38 (68)	
	Widowed	12 (28)	13 (23)	
	Single	1 (2)	5 (9)	
**Type of household, n (%)**				.03
	Alone	11 (26)	9 (16)	
	With spouse	30 (70)	34 (61)	
	With others	2 (5)	13 (23)	
**Subjective economic status, n (%)**				.25
	Low	10 (23)	8 (14)	
	Moderate	30 (70)	39 (70)	
	High	3 (7)	9 (16)	
**Previous employment status, n (%)^c^**				.73
	Yes	30 (70)	42 (75)	
	No	8 (19)	10 (18)	
Vision impairment (yes), n (%)		10 (23)	13 (23)	>.99
Hearing impairment (yes), n (%)		10 (23)	7 (13)	.16
**Subjective health status, n (%)^c^**				.18
	Poor	11 (26)	8 (14)	
	Fair	9 (21)	5 (9)	
	Good	9 (21)	18 (32)	
	Very good	11 (26)	15 (27)	
	Excellent	3 (7)	9 (16)	
**Number of medications, n (%)^c^**				.35
	≤2	12 (28)	25 (45)	
	3-5	15 (35)	17 (30)	
	≥6	14 (33)	12 (21)	
Cognitive function (K-MMSE-2^d^), mean (SD)		27.5 (1.8)	28.1 (1.6)	.10
Subjective Cognitive Decline (SCD-Q^e^), mean (SD)		8.1 (6.1)	5.8 (5.4)	.05
IADL disability (K-IADL^f^ score>10), n (%)		12 (28)	11 (20)	.33
**Frailty (FPQ^g^), n (%)**				.07
	Robust	20 (47)	36 (64)	
	Prefrailty	15 (35)	17 (30)	
	Frailty	8 (19)	3 (5)	
Depression (SGDS-K^h^), mean (SD)		5.6 (1.9)	5.1 (1.9)	.22
Anxiety (K-GAI^i^), mean (SD)		4.8 (5.7)	3.5 (4.6)	.19
Mild Behavioral Impairment (MBI-C^j^), mean (SD)		9.9 (10.1)	5.1 (6.2)	.01

^a^SCD: subjective cognitive decline.

^b^MCI: mild cognitive impairment.

^c^Some missing data.

^d^K-MMSE-2: Korean Mini-Mental State Examination, second edition.

^e^SCD-Q: Subjective Cognitive Decline Questionnaire.

^f^K-IADL: Korean Instrumental Activities of Daily Living.

^g^FPQ: Frailty Phenotype Questionnaire.

^h^SGDS-K: Korean version of the Short Form-Geriatric Depression Scale.

^i^K-GAI: Korean version of the Geriatric Anxiety Inventory.

^j^MBI-C: Mild Behavioral Impairment Checklist.

Table 2 presents the baseline characteristics based on levels of loneliness. The high loneliness group consisted of 37 (37%) individuals, while the low loneliness group included 62 (63%) individuals. The mean age in the low loneliness group was 77.1 (SD 5.9) years, compared with 76.4 (SD 6.1) years in the high loneliness group.

**Table 2:** Baseline characteristics of participants by levels of loneliness (N=99).

Variables			Low (n=62)		High (n=37)		*P* value
**Predementia stage, n (%)**							.36
	SCD^a^	44 (71)		23 (62)			
	MCI^b^	18 (29)		14 (38)			
**Sex, n (%)**							.43
	Men	25 (40)		12 (32)			
	Women	37 (60)		25 (68)			
**Age (years), n (%)**							.77
	<70	9 (15)		6 (16)			
	70-74	10 (16)		7 (19)			
	75-79	18 (29)		13 (35)			
	≥80	25 (40)		11 (30)			
**Educational level, n (%)**							.48
	≤Elementary school	17 (27)		12 (32)			
	Middle school	9 (15)		8 (22)			
	High school	18 (29)		6 (16)			
	≥College	18 (29)		11 (30)			
**Marital status, n (%)**							.83
	Married	43 (69)		25 (68)			
	Widowed	16 (26)		9 (24)			
	Single	3 (5)		3 (8)			
**Type of household, n (%)**							.04
	Alone	9 (15)		11 (30)			
	With spouse	40 (65)		24 (65)			
	With others	13 (21)		2 (5)			
**Subjective economic status, n (%)**							<.001
	Low	3 (5)		15 (41)			
	Moderate	52 (84)		17 (46)			
	High	7 (11)		5 (14)			
**Previous employment status, n (%)^c^**							.49
	Yes	46 (74)		26 (70)			
	No	12 (19)		6 (16)			
Vision impairment (yes), n (%)			13 (21)		10 (27)		.49
Hearing impairment (yes), n (%)			11 (18)		6 (16)		.85
**Subjective health status, n (%)^c^**							.60
	Poor	11 (18)		8 (22)			
	Fair	8 (13)		6 (16)			
	Good	15 (24)		12 (32)			
	Very good	17 (27)		9 (24)			
	Excellent	10 (16)		2 (5)			
**Number of medications, n (%)^c^**							.85
	≤2	24 (39)		13 (35)			
	3-5	18 (29)		14 (38)			
	≥6	17 (27)		9 (24)			
Cognitive function (K-MMSE-2^d^), mean (SD)			28.0 (1.6)		27.5 (1.8)		.10
Subjective Cognitive Decline (SCD-Q^e^), mean (SD)			6.3 (5.6)		7.6 (6.2)		.30
IADL disability (K-IADL^f^ score > 10), n (%)			15 (24)		8 (22)		.77
**Frailty (FPQ^g^), n (%)**							.26
	Robust	39 (63)		17 (46)			
	Prefrailty	17 (27)		15 (41)			
	Frailty	6 (10)		5 (14)			
Depression (SGDS-K^h^), mean (SD)			5.1 (1.5)		5.6 (2.5)		.19
Anxiety (K-GAI^i^), mean (SD)			3.2 (4.7)		5.5 (5.6)		.03
Mild Behavioral Impairment (MBI-C^j^), mean (SD)			6.0 (7.0)		9.1 (10.5)		.11

^a^SCD: subjective cognitive decline.

^b^MCI: mild cognitive impairment.

^c^Some missing data.

^d^K-MMSE-2: Korean Mini-Mental State Examination, second edition.

^e^SCD-Q: Subjective Cognitive Decline Questionnaire.

^f^K-IADL: Korean Instrumental Activities of Daily Living.

^g^FPQ: Frailty Phenotype Questionnaire.

^h^SGDS-K: Korean version of the Short Form-Geriatric Depression Scale.

^i^K-GAI: Korean version of the Geriatric Anxiety Inventory.

^j^MBI-C: Mild Behavioral Impairment Checklist.

### Performance Comparison for Exploratory Models

**Figure 4:**
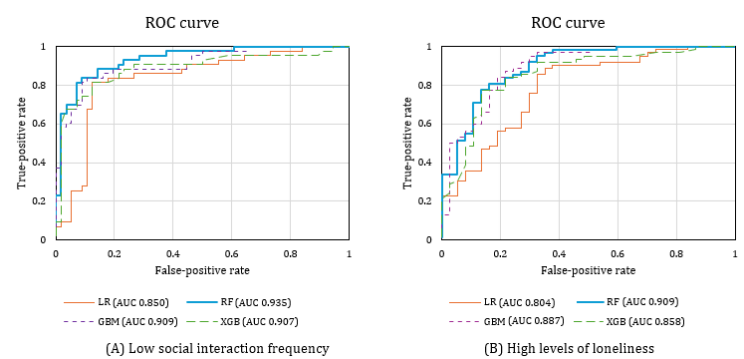
Receiver operator characteristic (ROC) curve for each model’s analysis of low social interaction frequency and high levels of loneliness. (A) ROC curve of the low social interaction frequency exploratory model. (B) ROC curve of the high levels of loneliness exploratory model. GBM: Gradient Boosting Machine; LR: logistic regression; RF: random forest; XGB: Extreme Gradient Boosting.

Table 3 and Figure 4 present the performance of the models exploring factors associated with low social interaction frequency and high levels of loneliness. Among the models, the RF model demonstrated the highest performance in identifying factors related to low social interaction frequency, while the GBM model showed the best performance in identifying factors related to high levels of loneliness. Specifically, the RF model achieved an AUC of 0.935, with macro and micro *F*_1_-scores of 0.824 and 0.828, respectively. By contrast, the GBM model yielded an AUC of 0.887, with a macro *F*_1_-score of 0.867 and a micro *F*_1_-score of 0.871.

**Table 3:** Performance comparison of the exploratory models for low social interaction frequency and high levels of loneliness^a^.

Exploration goal and model			AUC^b^		Accuracy		Precision		Specificity		*F*_1_-score		
											Macro^c^		Micro^d^
**Low social interaction frequency**													
	GBM^e^	0.909		0.850		0.837		0.857		0.829		0.828	
	LR^f^	0.850		0.780		0.837		0.732		0.763		0.766	
	*RF* ^g^	*0.935*		*0.849*		*0.837*		*0.857*		*0.824*		*0.828*	
	XGBoost^h^	0.907		0.840		0.814		0.857		0.814		0.814	
**High levels of loneliness**													
	*GBM*	*0.887*		*0.838*		*0.871*		*0.784*		*0.867*		*0.871*	
	LR	0.804		0.779		0.839		0.676		0.825		0.825	
	RF	0.909		0.818		0.871		0.730		0.854		0.857	
	XGBoost	0.858		0.798		0.839		0.730		0.838		0.839	

^a^The italicized values indicate the machine learning models with the best performance within each category (ie, low social interaction frequency and high levels of loneliness).

^b^AUC: area under the receiver operator characteristic curve.

^c^Average *F*_1_-score for 10 folds.

^d^Calculated as the sum of the confusion matrix of folds.

^e^GBM: Gradient Boosting Machine.

^f^LR: logistic regression.

^g^RF: random forest.

^h^XGBoost: Extreme Gradient Boosting.

### Feature Importance

Table 4 summarizes the key features identified by the RF model, which exhibited the best performance in identifying factors associated with low social interaction frequency. Among all features, physical movement emerged as the most influential variable, showing the highest feature importance. Given the relatively higher importance of actigraphy-derived features compared with demographic and health-related variables, the feature importance values for the survey-based data are presented separately for clarity (see Multimedia Appendix 4). Among the demographic and health-related variables, the total score across the MBI-C domains demonstrated the highest feature importance.

**Table 4:** The feature importance for exploring factors related to low social interaction frequency, as analyzed by 4 groups.

Variables	Feature importance
Physical movement	5.223
Sleep quantity	2.128
Sleep quality	0.795
Sedentary behavior	0.751
Sum of MBI-C^a^	0.103
Impulse dyscontrol domain in MBI-C	0.084
Sum of K-GAI^b^	0.053
Living with offspring	0.048
Sum of SCD-Q^c^	0.044
Sex	0.035

^a^MBI-C: Mild Behavioral Impairment Checklist.

^b^K-GAI: Korean version of the Geriatric Anxiety Inventory.

^c^SCD-Q: Subjective Cognitive Decline Questionnaire.

Table 5 presents the representative influential features associated with a high level of loneliness, as identified by the GBM model, which demonstrated the best performance for this outcome. Among all features, actigraphy data emerged as the most influential, with sleep quality showing the highest feature importance. Given the relatively greater influence of actigraphy-derived variables compared with survey-based data, the feature importance values for the survey data are provided separately for clarity (see Multimedia Appendix 5). When actigraphy features were excluded, the total score on the SGDS-K emerged as the most influential survey-based predictor.

**Table 5:** The feature importance for exploring factors related to high levels of loneliness, as analyzed by 4 groups.

Variables	Feature importance
Sleep quality	3.206
Physical movement	2.173
Sedentary behavior	1.723
Sleep quantity	0.812
Sum of SGDS-K^a^	0.143
Sum of SCD-Q^b^	0.128
Sum of K-GAI^c^	0.099
Sum of MBI-C^d^	0.069
Impulse dyscontrol domain in MBI-C	0.062
Subjective economic status	0.058

^a^SGDS-K: Korean version of the Short Form-Geriatric Depression Scale.

^b^SCD-Q: Subjective cognitive decline questionnaire.

^c^K-GAI: Korean Geriatric Anxiety Inventory.

^d^MBI-C: Mild behavioral impairment checklist.

## Discussion

### Principal Findings

This study developed and validated models that integrated objectively measured sleep and physical activity data from actigraphy, EMA responses, and self-reported survey data to investigate factors associated with social isolation among older adults in the predementia stage. The findings identified distinct factors linked to low social interaction frequency and high levels of loneliness, underscoring the multidimensional nature of social isolation and its underlying mechanisms. These results emphasize the value of combining subjective data from EMA with objective actigraphy-derived measures to better understand the complex factors associated with social isolation—key indicators of cognitive decline in older adults.

### Comparison With Previous Studies

This study sought to address the limitations of conventional tools for assessing social isolation. Existing measures, such as the Steptoe Social Isolation Index, rely on retrospective self-reports of social interactions over extended periods (eg, 1 month) [63], rendering them vulnerable to recall bias. Moreover, single–time-point assessments fail to capture the dynamic and fluctuating nature of social isolation [64], limiting their sensitivity in detecting subtle changes that may indicate elevated risk for cognitive decline. These methodological shortcomings impede the early and accurate identification of high-risk individuals in the predementia stage, thereby delaying timely and targeted interventions. Bridging these gaps is essential, as early detection and intervention for social isolation represent critical components of effective dementia prevention strategies.

To overcome these challenges, this study used EMA, which enables real-time and repeated evaluation of social isolation in naturalistic settings [23]. We focused on 2 core dimensions of social isolation—social interaction frequency and loneliness—to improve the precision of identifying high-risk individuals. By integrating subjective EMA data with objective actigraphy-derived measures, this study offered a more nuanced and comprehensive assessment of social isolation. This approach captured temporal variability and behavioral patterns that are often missed by traditional, single–time-point assessments.

Through EMA, participants reported their social experiences and emotional states in real time, while actigraphy provided objective measurements of physical activity and behavioral patterns that may not be consciously perceived [28-30]. This integrated approach addresses the limitations of traditional survey-based assessments by offering a multidimensional perspective on social isolation. By combining subjective self-reports with objective behavioral data, the study facilitated more accurate identification of high-risk individuals, thereby informing strategies for early and targeted intervention.

### Actigraphy Factors Related to Low Social Interaction Frequency

Among the factors associated with low social interaction, physical movement demonstrated the highest feature importance, suggesting a stronger potential link compared with sleep-related variables. This finding aligns with previous research emphasizing the pivotal role of physical activity in preserving mobility, which is fundamental for active social engagement. Physical activity contributes to maintaining pulmonary capacity, preventing frailty, and supporting overall physical function [65]. Notably, it is critical for sustaining mobility—the ability to move independently within one’s home, neighborhood, and broader community settings [66,67]. A decline in mobility can restrict engagement in daily activities and limit opportunities for social participation. A study that used structural equation modeling among Chinese older adults aged 65 and above found that mobility impairments were significantly associated with decreased levels of social participation [68]. These findings underscore the critical role of physical activity in maintaining mobility and suggest that reduced mobility may contribute to lower levels of social engagement.

Sleep may have a relatively weaker direct association with social interaction frequency compared with physical activity. However, previous studies suggest that sleep can significantly influence social experiences. One plausible explanation is that adequate sleep duration and better sleep quality can promote higher levels of physical activity [69,70], which in turn may indirectly enhance social interaction. Conversely, insufficient sleep has been linked to heightened emotional reactivity and increased negative emotional states, such as anxiety and depression [71], which are associated with impaired communication skills and reduced social engagement [72]. Moreover, poor sleep can diminish the desire to connect with others and reduce positive social emotions, further contributing to lower social interaction frequency [73].

Therefore, further research is warranted to investigate detailed sleep characteristics—such as sleep environment factors or brainwave patterns measured via electroencephalography—to better understand their influence on social interaction frequency and to deepen insights into this relationship.

### Actigraphy Factors Related to High Levels of Loneliness

Among the factors associated with high levels of loneliness, sleep quality demonstrated the greatest feature importance. The relationship between sleep quality and loneliness is complex and bidirectional [74]. Loneliness can impair sleep quality by increasing unconscious vigilance toward social threats [75], which act as potent triggers for cortisol release [76]. Elevated cortisol levels have been linked to higher sleep fragmentation scores [77], further contributing to poorer sleep quality. Although previous studies have emphasized the increased risk of declining sleep quality during the predementia stage [78], the mechanisms linking loneliness and sleep quality remain complex [79], warranting further investigation to elucidate these underlying processes. In light of this, our study uses objective measures such as actigraphy alongside momentary assessment methods to enable a more precise analysis of the relationship between loneliness and sleep quality. Consequently, our findings contribute to the development of targeted interventions focused on the early detection and prevention of high levels of loneliness.

In the exploratory model for loneliness, physical movement emerged as the second most important factor. While individuals experiencing loneliness often demonstrate lower levels of physical activity [80], some studies suggest that the association between physical movement and loneliness remains uncertain and generally unclear [81]. The inconsistent findings in previous research regarding the relationship between physical activity and loneliness suggest that the quality of social relationships may moderate this association. Systematic reviews indicate that the quality of social relationships plays a significant role in the link between physical activity and loneliness [82], implying that merely increasing physical activity may not be sufficient to fully explain or address changes in loneliness.

From this perspective, integrating physical activity with social interaction frequency may offer a more effective approach to understanding loneliness. For instance, group-based physical activities that incorporate social engagement can foster the formation of new social networks and strengthen social connectedness [83]. This dual benefit not only promotes increased physical activity but also provides opportunities to enhance the quality of social relationships, ultimately contributing to the alleviation of loneliness. Additionally, given that individuals experiencing loneliness may be more hesitant to engage in physical activity [82], the social support inherent in group-based physical activities can serve as a motivating factor, positively impacting the reduction of loneliness [84]. Thus, an approach that integrates both physical activity and social interaction may be crucial for elucidating the relationship between physical activity and loneliness.

### Survey Data Related to Low Social Interaction Frequency and High Levels of Loneliness

Among the survey data, low social interaction frequency was most strongly associated with Korean version MBI-C scores, while high levels of loneliness were more closely related to SGDS-K scores. This distinction suggests that low social interaction frequency is primarily linked to behavioral factors, whereas high levels of loneliness are more closely associated with emotional states. There were limitations in directly comparing Korean version MBI-C scores between groups with low and high social interaction frequencies using the ML model. However, descriptive analysis revealed that the low social interaction frequency group exhibited higher MBI-C scores compared with the high social interaction frequency group (Table 1), indicating more severe behavioral symptoms. One symptom of MBI includes emotional dysregulation, characterized by feelings of despair, sadness, and worry [54]. Emotional regulation significantly influences interpersonal interactions and plays a crucial role in maintaining relationships. Consequently, difficulties in managing emotions can lead to challenges in social behavior [85]. These findings suggest that MBI symptoms may be more pronounced in older adults with low social interaction frequency, underscoring the importance of identifying these individuals to better understand patterns of social interaction. Moreover, they highlight the need for further research to explore the complex mechanisms linking MBI symptoms and social interaction frequency.

Although it was difficult to establish the directionality of SGDS-K scores in relation to high levels of loneliness using both ML models and descriptive analysis (Table 2), SGDS-K scores nonetheless emerged as a significant factor associated with heightened loneliness. Older adults experiencing depression are at greater risk of adverse psychological effects and tend to be more vulnerable to feelings of loneliness [86]. Depression and loneliness share a bidirectional relationship [87] and are both closely linked to white matter integrity in the brain [88]. Increased mean diffusivity in white matter has been associated with greater levels of loneliness and depression [89,90]. Early identification of depression in older adults at the predementia stage, along with targeted interventions, may help alleviate loneliness. Further research is warranted to clarify the complex interactions among these factors.

### Comparison Factors Related to Low Social Interaction Frequency and High Levels of Loneliness

This study demonstrated that social interaction frequency—an objective dimension of social isolation—and loneliness—its subjective counterpart—may be influenced by different factors. The findings indicate that key factors associated with low social interaction frequency differ from those linked to high levels of loneliness. This distinction underscores that social isolation is a multidimensional construct, with social interaction frequency and loneliness potentially shaped by distinct underlying mechanisms. Therefore, addressing social isolation requires a nuanced approach that acknowledges the unique characteristics of social interaction frequency and loneliness, rather than relying on a one-size-fits-all intervention strategy. Based on these findings, future research should focus on developing tailored interventions that specifically target the distinct aspects of social interaction frequency and loneliness.

### Limitations and Strengths

Our study has several limitations. First, the cross-sectional design limits our ability to fully elucidate complex relationships, such as causal pathways among sleep, social interaction frequency, and loneliness. While our findings highlight significant associations with social isolation, it remains unclear whether these factors act as antecedents or consequences of social isolation. Given the interconnected nature of sleep, social interaction frequency, and loneliness [74], future research should employ longitudinal tracking to better understand the temporal and potentially causal relationships among these factors. Second, the limited regional scope and relatively small sample size constrain the generalizability of our findings. Expanding studies to include more diverse populations and larger cohorts will be necessary to validate and extend these results. Considering that sensitivity to social isolation may vary depending on whether individuals belong to individualistic or collectivist cultures [91], future research should include diverse regions and larger samples to explore cultural differences in sensitivity to social isolation. Third, the method we used to classify the 3 clusters into low and high groups for social interaction frequency and loneliness may not have fully captured the qualitative aspects of social interaction that could have influenced these classifications. This study utilized EMA for these classifications. Although recent research on populations with cognitive impairment has highlighted the feasibility and utility of EMA, it has not yet been fully validated. Therefore, the results of these group classifications should be interpreted with caution. Future studies are recommended to build on the current findings to strengthen the validity of EMA and to adopt tools such as cross tables to develop more refined and precise classifications of social interaction frequency and loneliness.

Fourth, given that SCD and MCI represent different levels of cognitive impairment, they may have distinct associations with social interaction frequency and loneliness. However, due to the limited sample size, we were unable to perform subgroup analyses. Future research should address this limitation by recruiting a larger sample and conducting separate analyses for each group to better understand their unique characteristics. Lastly, the approach of categorizing social interaction frequency and loneliness levels as high or low based on averages in this study did not directly capture variability or stability. To reduce potential information loss caused by daily averages, we segmented the day into 4 distinct periods (night, morning, afternoon, and evening). This segmentation was based on the activity characteristics of the Korean population [41] and indirectly captured variability and stability. Additionally, we utilized high-density actigraphy data collected continuously over 24 hours to gain a deeper understanding of the temporal characteristics of each group within these defined periods. Given that the primary objective of this study was to provide foundational insights and contribute to the development of subsequent research, it represents an early-stage effort to comprehensively explore factors related to social interaction frequency and loneliness through the integration of subjective data (eg, EMA) and objective data (eg, actigraphy).

Despite several limitations, this study has multiple strengths. First, it developed models to explore factors related to both low social interaction frequency and high levels of loneliness, which are negatively associated with cognitive function in individuals at risk for dementia, such as older adults with predementia. Specifically, by examining social interaction frequency and loneliness in real time, the study minimizes recall bias and offers more accurate insights. Additionally, it identifies objective factors related to low social interaction frequency and high levels of loneliness using quantitative actigraphy data, thereby providing key elements for addressing these issues.

Second, this study contributes to understanding the nuanced aspects of social isolation in the predementia population by highlighting that different factors are associated with low social interaction frequency and high levels of loneliness. Although social interaction frequency and loneliness are closely related aspects of social isolation, they may operate through distinct mechanisms. This analysis provides a valuable perspective on their potential differences by demonstrating the varying importance of shared factors and identifying unique influences on each aspect. Furthermore, it suggests that intervention strategies aimed at reducing social isolation should avoid a one-size-fits-all approach and instead be tailored to address the specific characteristics of social interaction frequency and loneliness separately.

This study demonstrated the potential of an ML-based exploratory model to identify vulnerable groups among older adults with SCD and MCI, based on the frequency of social interactions and levels of loneliness, using data collected from mobile EMA and wearable actigraphy. The findings may inform interventions aimed at reducing the frequency of social isolation and, ultimately, contribute to dementia prevention in older adults at high risk of cognitive impairment.

### Conclusions

This study demonstrated the potential of ML-based predictive models to explore factors related to social interaction frequency and loneliness levels, leveraging data collected through mobile EMA, wearable actigraphy, and survey responses. The findings also suggest that the objective aspect of social isolation—represented by social interaction frequency—and the subjective aspect—represented by loneliness—may operate through distinct mechanisms. These results have clinical implications for developing digital interventions to mitigate social isolation in older adults with predementia, ultimately helping to prevent cognitive decline in this at-risk population.

## Appendix

Multimedia Appendix 1 Detailed description of the deep embedding clustering method.

Multimedia Appendix 2 Determination of optimal number of clusters.

Multimedia Appendix 3 Classification of high and low levels of social interaction frequency and loneliness groups.

Multimedia Appendix 4 Feature importance for low social interaction frequency in survey data.

Multimedia Appendix 5 Feature importance for high levels of loneliness in survey data.

## Abbreviations

AUC: area under the receiver operating characteristic curve
DEC: Deep Embedding Clustering
EMA: ecological momentary assessment
GBM: Gradient Boosting Machine
ICC: intraclass correlation coefficient
K-GAI: Korean version of the Geriatric Anxiety Inventory
K-IADL: Korean Instrumental Activities of Daily Living
K-MMSE: Korean Mini-Mental State Examination
MCI: mild cognitive impairment
ML: machine learning
RF: random forest
RN: registered nurse
SCD: subjective cognitive decline
SCD-Q: Subjective Cognitive Decline Questionnaire
SGDS-K: Korean version of the Short Form Geriatric Depression Scale
TST: total sleep time
WASO: wake after sleep onset
XGBoost: Extreme Gradient Boosting

## Data Availability

The data sets used or analyzed in this study are available from the first author (BK) upon reasonable request.
