# Supplementary material for: Exploring Factors Related to Social Isolation Among Older Adults in the Predementia Stage Using Ecological Momentary Assessments and Actigraphy: Machine Learning Approach
Source: J Med Internet Res. 2025 Jun 23;27:e69379. doi: 10.2196/69379 (PMC12235200; doi:10.2196/69379)
Supplement: Multimedia Appendix 1 [file jmir_v27i1e69379_app1.docx]

We implemented the Deep Embedding Clustering (DEC) algorithm to learn meaningful low-dimensional representations of high-dimensional EMA data and identify latent subgroups of participants based on their social interaction and loneliness profiles. The DEC model was trained and evaluated using the following architecture, hyperparameter training procedures:

**1. Network Architecture**

1.1. Encoder

The network architecture includes four main components: an encoder, a bottleneck layer, a decoder, and a clustering layer. First, the encoder is composed of a 3-layer 2D convolutional structure with progressively increasing kernel sizes: 48 kernels in the first layer, 96 in the second, and 192 in the third.

1.2. Bottleneck

Next, the bottleneck is a single 2D convolutional layer with 32 kernels, serving as the latent embedding space that captures compressed feature representations.

1.3. Decoder

The decoder mirrors the encoder with a symmetric 2D convolutional architecture, reconstructing the input from the latent space using layers with 192, 96, and 48 kernels in reverse order.

1.4. Clustering Layer

Following the decoder, the extracted latent features are passed into a clustering layer, where the number of clusters k is pre-specified within the range of 2 to 10.

All convolutional layers in the encoder, bottleneck, and decoder utilize kernels with a window size of 1 and a kernel size of 20, and they employ the ReLU activation function with Glorot normal initialization.

**2. Hyperparameter Settings**

2.1. Neural Network parameters

The neural network was trained using the following key parameters, selected to ensure stable learning and convergence: 100 epochs, a batch size of 8, and a learning rate of 0.001. The tolerance for convergence was set to 0.001, the maximum number of iterations was 2000, and the update interval for clustering was set to 250.

2.2. Clustering parameters

For the clustering parameters, the number of clusters k was set to range from 2 to 10 to explore the optimal number of clusters for the model.

**3. Training Process**

3.1 Pretraining Phase

The encoder, bottleneck, and decoder were pretrained using mean squared error (MSE) to minimize the reconstruction loss between the original input and the reconstructed output.

3.2: Clustering Optimization Phase

After pretraining, the latent embeddings were input into the clustering layer. The model iteratively refined cluster assignments by minimizing the Kullback–Leibler (KL) divergence between the soft cluster assignment distribution and an auxiliary target distribution.

3.2.1. Loss functions

Different loss functions were applied to each training phase to optimize reconstruction and clustering performance. For the autoencoder structure, including the encoder, bottleneck, and decoder, we employed the Mean Squared Error (MSE) as the loss function. In the clustering phase, we used the Kullback–Leibler (KL) divergence.

3.2.2. Optimizer

The optimization algorithm applied to the encoder, bottleneck, and decoder was the Adam optimizer, while the clustering step utilized Stochastic Gradient Descent (SGD).

3.2.3. Metrics

Model performance was evaluated using MSE for the autoencoder and clustering accuracy for the final cluster assignments.
