# Supplementary material for: Exploring Factors Related to Social Isolation Among Older Adults in the Predementia Stage Using Ecological Momentary Assessments and Actigraphy: Machine Learning Approach
Source: J Med Internet Res. 2025 Jun 23;27:e69379. doi: 10.2196/69379 (PMC12235200; doi:10.2196/69379)
Supplement: Multimedia Appendix 2 [file jmir_v27i1e69379_app2.docx]

Determination of optimal number of clusters for social interaction frequency and levels of loneliness using silhouette coefficient and WCSS.


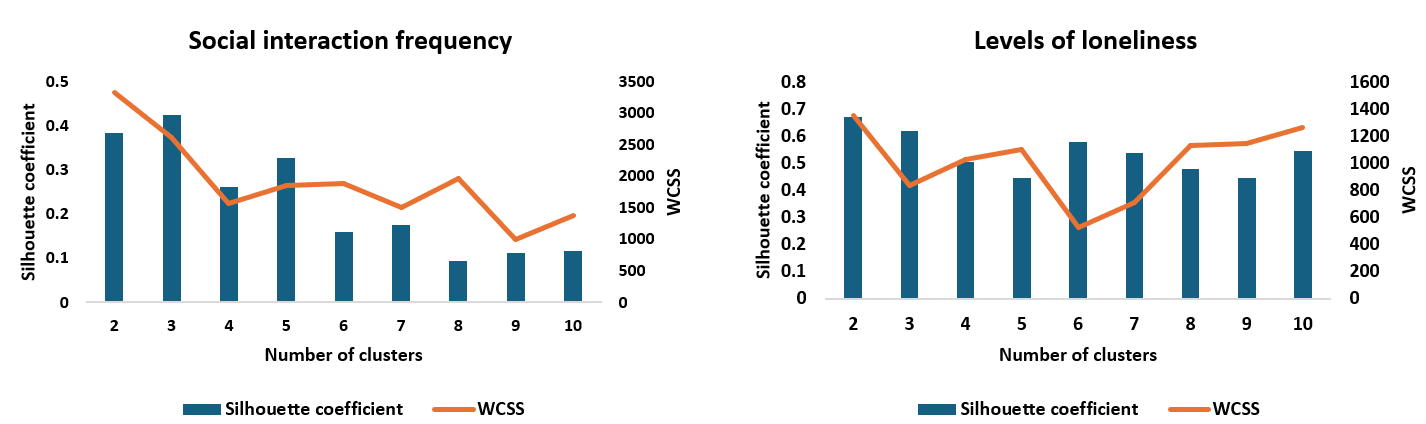


WCSS: within-cluster sum of squares
