# Supplementary material for: Exploring Factors Related to Social Isolation Among Older Adults in the Predementia Stage Using Ecological Momentary Assessments and Actigraphy: Machine Learning Approach
Source: J Med Internet Res. 2025 Jun 23;27:e69379. doi: 10.2196/69379 (PMC12235200; doi:10.2196/69379)

Classification of high and low social interaction frequency groups based on the two embedding vectors generated by the Autoencoder.


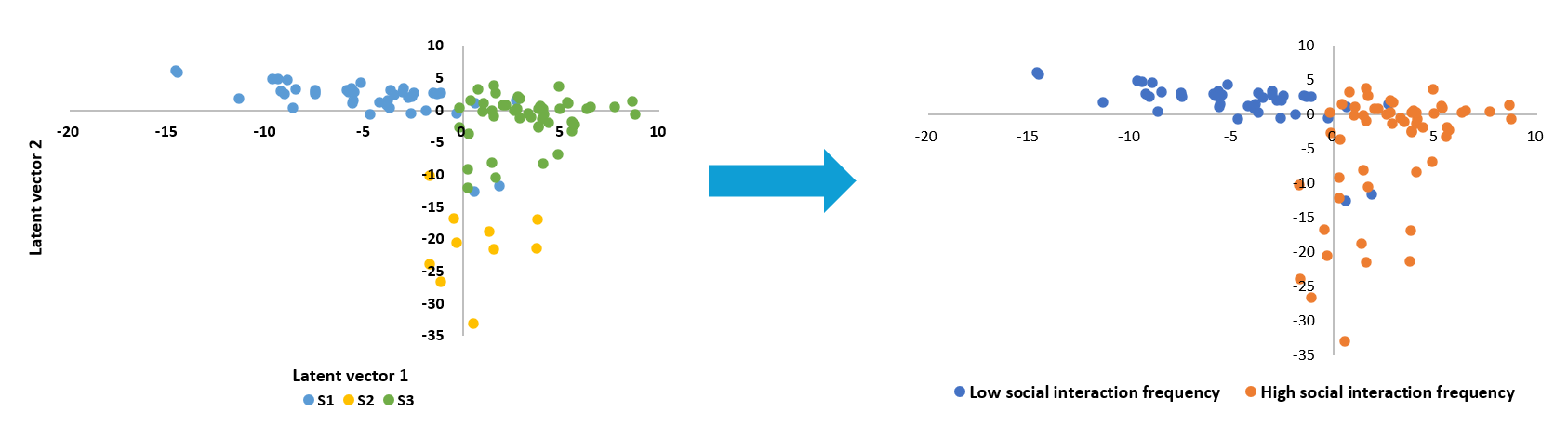


Classification of high and low levels of loneliness groups based on the two embedding vectors generated by the Autoencoder.


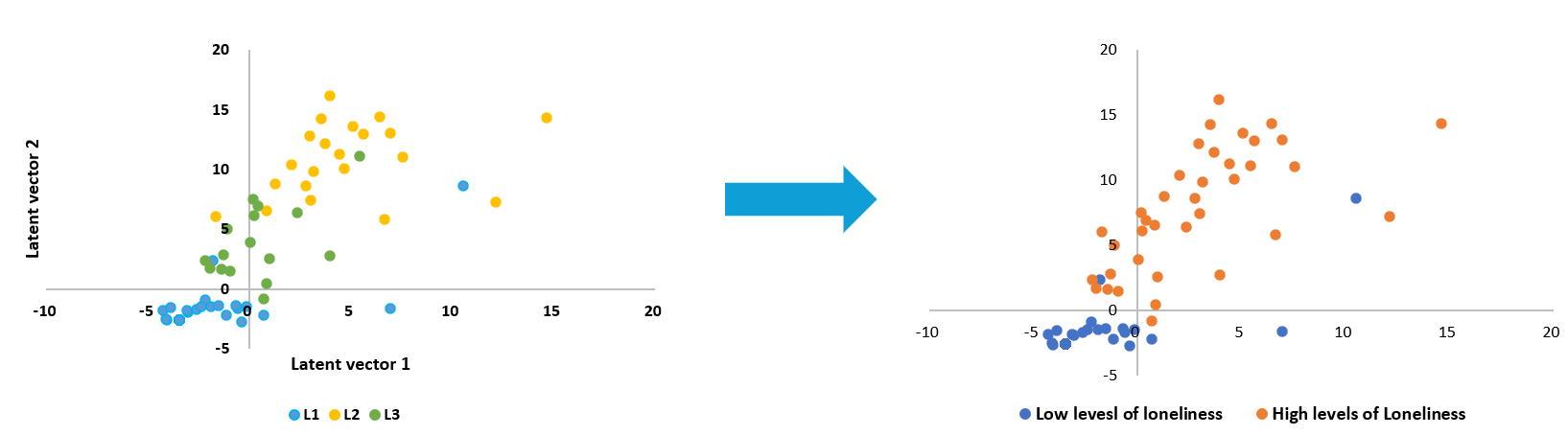

Supplement: Multimedia Appendix 3 [file jmir_v27i1e69379_app3.docx]
