# Supplementary material for: Exploring Factors Related to Social Isolation Among Older Adults in the Predementia Stage Using Ecological Momentary Assessments and Actigraphy: Machine Learning Approach
Source: J Med Internet Res. 2025 Jun 23;27:e69379. doi: 10.2196/69379 (PMC12235200; doi:10.2196/69379)
Supplement: Multimedia Appendix 4 [file jmir_v27i1e69379_app4.docx]

Feature importance exploring factors related to low levels of social interaction frequency in survey data derived from the original analysis.

| **Variables** | **Feature importance** |
| --- | --- |
| Sum of MBI-C^a^ | 0.103 |
| Impulse dyscontrol domain in MBI-C | 0.084 |
| Sum of K-GAI^b^ | 0.053 |
| Living with offspring | 0.048 |
| Sum of SCD-Q^c^ | 0.044 |
| Sex | 0.035 |
| Sum of SGDS-K^d^ | 0.016 |
| Age | 0.011 |
| Educational level | 0.008 |
| Gastrointestinal disorder | 0.008 |

^a^MBI-C: Mild Behavioral Impairment Checklist

^b^K-GAI: Korean version of Geriatric Anxiety Inventory

^c^SCD-Q: Subjective Cognitive Decline Questionnaire

^d^K-GAI: Korean version of Geriatric Anxiety Inventory
