# Supplementary material for: Exploring Factors Related to Social Isolation Among Older Adults in the Predementia Stage Using Ecological Momentary Assessments and Actigraphy: Machine Learning Approach
Source: J Med Internet Res. 2025 Jun 23;27:e69379. doi: 10.2196/69379 (PMC12235200; doi:10.2196/69379)
Supplement: Multimedia Appendix 5 [file jmir_v27i1e69379_app5.docx]

Feature importance exploring factors related to high levels of loneliness in survey data derived from the original analysis.

| **Variables** | **Feature importance** |
| --- | --- |
| Sum of SGDS-K^a^ | 0.143 |
| Sum of SCD-Q^b^ | 0.128 |
| Sum of K-GAI^c^ | 0.099 |
| Sum of MBI-C^d^ | 0.069 |
| Impulse dyscontrol domain in MBI-C | 0.062 |
| Subjective economic status | 0.058 |
| Living alone | 0.024 |
| Musculoskeletal disorders | 0.024 |
| Drinking | 0.023 |
| Previous employment duration | 0.022 |

^a^SGDS-K: Korean version of Short Form-Geriatric Depression Scale

^b^SCD-Q: Subjective cognitive decline questionnaire

^c^K-GAI: Korean Geriatric Anxiety Inventory

^d^MBI-C: Mild behavioral impairment checklist.
